# Correction: Potential evidence of reengagement attempts following interruptions of a triadic social game in bonobos and chimpanzees

**DOI:** 10.1371/journal.pone.0338981

**Published:** 2025-12-12

**Authors:** The PLOS One Staff

## Notice of Republication

This article was republished on November 24, 2025, to correct Tables 2 and 3 because their legends were inverted. Please download this article again to view the correct version. The originally published, uncorrected article and the republished, corrected article are provided here for reference.

## Supporting information

S1 File Originally published, uncorrected article. (PDF)

S2 File Republished, corrected article. (PDF)
