# Supplementary material for: Potential evidence of reengagement attempts following interruptions of a triadic social game in bonobos and chimpanzees
Source: PLoS One. 2025 Mar 26;20(3):e0292984. doi: 10.1371/journal.pone.0292984 (PMC11940663; doi:10.1371/journal.pone.0292984)
Supplement: S1 Text — (DOCX) [file pone.0292984.s001.docx]

# S1 Text

We additionally attempted two conditions, which were randomly administered alongside other conditions. These conditions had to be discarded post-hoc from the main analyses due to methodological limitations. In the third attempted condition (“clumsy request”), conducted in all groups, the experimenter was supposed to simulate being clumsy, by reaching to the hose with the hand but not being “able” to properly grab it. The problem was that the hose could either stick out through the cage mesh, reachable for the experimenter, or it could fall within the cage, inaccessible for the experimenter. The clumsy request thus could have been perceived by the apes based on different intentions in these two cases; in the first, the experimenter could have been perceived as willing but unskilful (but could potentially grab the hose), and in the second, the experimenter could have been perceived as willing but incapable (could never grab the hose even if willing to do so). Overall, we attempted the “clumsy request” condition on 59 occasions, yet given the inconsistencies between experimenters and trials, we excluded this condition from our analysis (10 times in infant bonobos; 13 times in adult bonobos; 36 times in chimpanzees). In a fourth attempted condition (“third-party interruption”), only applied to adult and infant bonobos, the experimenter was interrupted by another person, who approached and started to talk to the experimenter. This condition was mainly implemented in bonobos and contained errors due to difficulty in implementation and a very small sample size; it shall be repeated by standardizing experimenter’s behaviours, since the experimenters often made mistakes, leading to inconsistencies in the test. For instance, they were either a) turning too slowly toward the third person who interrupts, b) holding the hose for too long while already starting to talk to the third person, c) not engaging fully in the conversation with third person, or d) turning around to check, due to other conflicts in the group of bonobos, or other disturbing bonobos. We attempted this condition 26 times (9 times in infant bonobos; 15 times in adult bonobos; 2 times in chimpanzees).
