# Supplementary material for: Potential evidence of reengagement attempts following interruptions of a triadic social game in bonobos and chimpanzees
Source: PLoS One. 2025 Mar 26;20(3):e0292984. doi: 10.1371/journal.pone.0292984 (PMC11940663; doi:10.1371/journal.pone.0292984)
Supplement: S1 Table — (DOCX) [file pone.0292984.s002.docx]

**S1 Table.** Information about study subjects. Ages for infant bonobos were estimated, as precise birthdates of orphans were unknown. Mo.-reared = Mother-reared.

| Subject (abbreviated ID) | Species | Site | Age (years) | Age category (this study) | Age class (Kano, 1992) | Sex | Rearing (for sanctuary  bonobos) |
| --- | --- | --- | --- | --- | --- | --- | --- |
| BA | Bonobo | Lola Ya Bonobo | 3.0 | Infant | Infant | Male | Orphan |
| BI | Bonobo | Lola Ya Bonobo | 4.0 | Infant | Infant | Male | Orphan |
| KW | Bonobo | Lola Ya Bonobo | 2.5 | Infant | Infant | Male | Orphan |
| LA | Bonobo | Lola Ya Bonobo | 4.5 | Infant | Infant | Female | Orphan |
| LU | Bonobo | Lola Ya Bonobo | 2.5 | Infant | Infant | Female | Orphan |
| BO | Bonobo | Lola Ya Bonobo | 9.0 | Adult | Subadult | Female | Orphan |
| EL | Bonobo | Lola Ya Bonobo | 6.0 | Adult | Juvenile | Female | Mo.-reared |
| IS | Bonobo | Lola Ya Bonobo | 21.0 | Adult | Adult | Female | Orphan |
| KE | Bonobo | Lola Ya Bonobo | 25.0 | Adult | Adult | Male | Orphan |
| KI | Bonobo | Lola Ya Bonobo | 8.0 | Adult | Subadult | Female | Orphan |
| LI | Bonobo | Lola Ya Bonobo | 8.0 | Adult | Subadult | Female | Mo.-reared |
| LO | Bonobo | Lola Ya Bonobo | 11.0 | Adult | Subadult | Male | Orphan |
| LUB | Bonobo | Lola Ya Bonobo | 6.0 | Adult | Juvenile | Female | Orphan |
| MI | Bonobo | Lola Ya Bonobo | 8.0 | Adult | Subadult | Female | Orphan |
| MO | Bonobo | Lola Ya Bonobo | 11.0 | Adult | Subadult | Male | Mo.-reared |
| OP | Bonobo | Lola Ya Bonobo | 23.0 | Adult | Adult | Female | Orphan |
| SI | Bonobo | Lola Ya Bonobo | 10.0 | Adult | Subadult | Male | Orphan |
| TC | Bonobo | Lola Ya Bonobo | 30.0 | Adult | Adult | Female | Orphan |
| CO | Chimpanzees | La Vallée des Singes | 22.0 | Adult | Adult | Male | - |
| JO | Chimpanzees | La Vallée des Singes | 23.0 | Adult | Adult | Male | - |
| LL | Chimpanzees | La Vallée des Singes | 9.0 | Adult | Subadult | Female | - |
| RO | Chimpanzees | La Vallée des Singes | 21.0 | Adult | Adult | Male | - |
| WO | Chimpanzees | La Vallée des Singes | 21.0 | Adult | Adult | Male | - |
