# Supplementary material for: Potential evidence of reengagement attempts following interruptions of a triadic social game in bonobos and chimpanzees
Source: PLoS One. 2025 Mar 26;20(3):e0292984. doi: 10.1371/journal.pone.0292984 (PMC11940663; doi:10.1371/journal.pone.0292984)
Supplement: S2 Table — (DOCX) [file pone.0292984.s003.docx]

**S2 Table.** Ethogram of signals (i.e., gestures, vocalizations, facial expressions) and GRBs after (1–6).

| **Type** | **Description** |
| --- | --- |
|  |  |
| **Gestures** | |
| Bang cage | Hand(s) (or feet) brought into hard audible contact with the cage mesh (can be repetitive) |
| Bipedal swagger | Side to side of forward and back movement while standing/walking bipedal (rarely also quadrupedal) |
| Dangle | Subject hangs from arm(s) from the top of the cage/mesh, with feet shaking loosely |
| Grab experimenter | Hand(s) firmly closed over part of experimenter’s body |
| Hit object | Hand(s) brought into short hard contact with the surface of an object |
| Hit object with object | Object brought into short hard contact with another object |
| Jump | While in bipedal position both feet leave ground simultaneously with horizontal displacement |
| Knock mesh/object | Back of hand/knuckles brought into short hard audible contact with cage mesh/object (can be repetitive) |
| Move hose | Hose is displaced in one direction, contact with hose is maintained throughout |
| Present body | Body part moved to deliberately expose an area to experimenter’s attention |
| Present genitals | Genitals moved to deliberately expose them to experimenter’s attention |
| Reach | Arm is extended to the experimenter with hand in open, palm exposed position |
| Shake experimenter | Hand(s) is firmly closed over part of experimenter’s body with repeated back and forth motion of hand |
| Shake head | Head moved back and forth in small, repeated side motion |
| Shake hose | Hand(s) firmly closed over hose with repeated up and down or side movements |
| Shake hand | Small repeated back and forth motion of hand(s) from wrist |
| Stomp | Sole of the foot/feet brought into short hard audible contact with surface |
| Touch experimenter | Palm of hand and/or body is brought to light contact with part of experimenter’s body, for under 2 s. Also counted if subject attempts to touch experimenter through the mesh but fingers are too short to reach experimenter’s body |
| Touch hose | Palm of hand and/or body is brought to light contact with hose, for under 2 s |
| **GRBs** | |
| Drop hose out of cage | Hose pushed through the mesh outside of cage and out of reach (from subject) |
| Hand back hose | One end of hose pushed through the cage mesh outside of cage, while hand is firmly closed over other end of hose |
| Prompt | Body/hand is moved in rapid, short, and tense movements, while hand is firmly closed over hose and while looking at experimenter |
| Simulate game action | Hose pushed back and forth through the mesh (mimicking the game’s pull and relax movements) |
| Touch with hose | Hose pushed through the cage mesh outside of cage and brought to light contact over part of experimenter’s body |
| **Vocalizations** | |
| Laughter | Voiceless breathing sounding like low-pitched grunting |
| Hoo | Quiet, relatively inconspicuous vocalization sounding like «*hoo*», mostly emitted as a single call |
| Peep | High-frequency, often closed-mouth vocalization; short in duration and flat, simple acoustic structure |
| **Facial expressions** | |
| Pout face | Lips pursed forward and curled outward in front (circular opening); lips pressed together at mouth corners |
| Tightened lips | Lips horizontally tensed; drawn inward or slightly baring teeth |
| Play face | Mouth opened with lips either in relaxed position covering the upper teeth completely and the lower teeth partially, or retracted, showing both upper and lower frontal teeth |

**References**

1. Byrne RW, Cartmill E, Genty E, Graham KE, Hobaiter C, Tanner J. Great ape gestures: intentional communication with a rich set of innate signals. Animal Cognition. 2017;20(4):755–69.

2. Clay Z, Archbold J, Zuberbühler K. Functional flexibility in wild bonobo vocal behaviour. PeerJ. 2015;3:e1124.

3. Crockford C, Gruber T, Zuberbühler K. Chimpanzee quiet hoo variants differ according to context. Royal Society Open Science. 2018 May 23;5(5):172066.

4. de Waal FBM. The communicative repertoire of captive bonobos (*Pan paniscus*), compared to that of chimpanzees. Behavior. 1988;106(3):183–251.

5. Genty E, Clay Z, Hobaiter C, Zuberbühler K. Multi-modal use of a socially directed call in bonobos. PloS one. 2014;9(1):e84738.

6. Genty E, Neumann C, Zuberbühler K. Complex patterns of signalling to convey different social goals of sex in bonobos, Pan paniscus. Scientific reports. 2015;5(16135):1–13.
