# Supplementary material for: Potential evidence of reengagement attempts following interruptions of a triadic social game in bonobos and chimpanzees
Source: PLoS One. 2025 Mar 26;20(3):e0292984. doi: 10.1371/journal.pone.0292984 (PMC11940663; doi:10.1371/journal.pone.0292984)
Supplement: S3 Table — (DOCX) [file pone.0292984.s004.docx]

**S3 Table.** Count of signals and game-related behaviors (GRB) used to reengage the partner across groups.

| **Reengagement type** | **Signal/GRB**  **type** | **Infant bonobos** | **Adult bonobos** | **Adult chimpanzees** |
| --- | --- | --- | --- | --- |
| Gesture | Bang cage | 0 | 0 | 1 |
| Gesture | Bipedal swagger | 0 | 0 | 2 |
| Gesture | Dangle | 2 | 0 | 0 |
| Gesture | Grab experimenter | 5 | 0 | 0 |
| Gesture | Hit object | 1 | 0 | 0 |
| Gesture | Hit object with object | 1 | 4 | 1 |
| Gesture | Jump | 1 | 0 | 0 |
| Gesture | Knock mesh | 0 | 0 | 2 |
| Gesture | Move hose | 3 | 0 | 0 |
| Gesture | Present body | 5 | 1 | 1 |
| Gesture | Present genitals | 1 | 2 | 0 |
| Gesture | Reach | 4 | 0 | 4 |
| Gesture | Shake experimenter | 2 | 0 | 0 |
| Gesture | Shake hand | 0 | 2 | 0 |
| Gesture | Shake head | 1 | 1 | 1 |
| Gesture | Shake hose | 3 | 8 | 5 |
| Gesture | Stomp | 0 | 0 | 3 |
| Gesture | Touch experimenter | 3 | 0 | 1 |
| Gesture | Touch hose | 0 | 1 | 2 |
| GRB | Hand back hose | 1 | 52 | 13 |
| GRB | Drop hose out of cage | 0 | 6 | 7 |
| GRB | Prompting | 0 | 5 | 6 |
| GRB | Simulate game action | 0 | 3 | 10 |
| GRB | Touch experimenter with hose | 1 | 2 | 9 |
| Facial expression | Play face | 2 | 3 | 0 |
| Facial expression | Pout face | 0 | 2 | 1 |
| Facial expression | Tightened lips | 0 | 0 | 1 |
| Vocalization | Hoo | 0 | 0 | 1 |
| Vocalization | Laughter | 0 | 1 | 0 |
| Vocalization | Peep | 0 | 1 | 0 |
