# Supplementary material for: Potential evidence of reengagement attempts following interruptions of a triadic social game in bonobos and chimpanzees
Source: PLoS One. 2025 Mar 26;20(3):e0292984. doi: 10.1371/journal.pone.0292984 (PMC11940663; doi:10.1371/journal.pone.0292984)
Supplement: S1 Data [file pone.0292984.s005.pdf]

Supplementary Information

1 S1 Data

The datasets of this study can be found on figshare.com: https://doi.org/10.6084/m9.figshare.21175129.v13

2 S1 Text

We additionally attempted two conditions, which were randomly administered alongside other conditions. These conditions had to be discarded post-hoc from the main analyses due to methodological limitations.

In the third attempted condition (“clumsy request”), conducted in all groups, the experimenter was supposed to simulate being clumsy, by reaching to the hose with the hand but not being “able” to properly grab it. The problem was that the hose could either stick out through the cage mesh, reachable for the experimenter, or it could fall within the cage, inaccessible for the experimenter. The clumsy request thus could have been perceived by the apes based on different intentions in these two cases; in the first, the experimenter could have been perceived as willing but unskilful (but could potentially grab the hose), and in the second, the experimenter could have been perceived as willing but incapable (could never grab the hose even if willing to do so). Overall, we attempted the “clumsy request” condition on 59 occasions, yet given the inconsistencies between experimenters and trials, we excluded this condition from our analysis (10 times in infant bonobos; 13 times in adult bonobos; 36 times in chimpanzees).

In a fourth attempted condition (“third-party interruption”), only applied to adult and infant bonobos, the experimenter was interrupted by another person, who approached and started to talk to the experimenter. This condition was mainly implemented in bonobos and contained errors due to difficulty in implementation and a very small sample size; it shall be repeated by standardizing experimenter’s behaviours, since the experimenters often made mistakes, leading to inconsistencies in the test. For instance, they were either a) turning too slowly toward the third person who interrupts, b) holding the hose for too long while already starting to talk to the third person, c) not engaging fully in the conversation with third person, or d) turning around to check, due to other conflicts in the group of bonobos, or other disturbing bonobos. We attempted this condition 26 times (9 times in infant bonobos; 15 times in adult bonobos; 2 times in chimpanzees).

S1 Table. Information about study subjects. Ages for infant bonobos were estimated, as precise birthdates of orphans were unknown. Mo.-reared = Mother-reared.

**Subject (abbreviated ID)**	**Species**	**Site**	**Age (years)**	**Age category (this study)**	**Age class** (Kano, 1992)	**Sex**	**Rearing (for sanctuary bonobos)**
BA	Bonobo	Lola Ya Bonobo	3.0	Infant	Infant	Male	Orphan
BI	Bonobo	Lola Ya Bonobo	4.0	Infant	Infant	Male	Orphan
KW	Bonobo	Lola Ya Bonobo	2.5	Infant	Infant	Male	Orphan
LA	Bonobo	Lola Ya Bonobo	4.5	Infant	Infant	Female	Orphan
LU	Bonobo	Lola Ya Bonobo	2.5	Infant	Infant	Female	Orphan
BO	Bonobo	Lola Ya Bonobo	9.0	Adult	Subadult	Female	Orphan
EL	Bonobo	Lola Ya Bonobo	6.0	Adult	Juvenile	Female	Mo.-reared
IS	Bonobo	Lola Ya Bonobo	21.0	Adult	Adult	Female	Orphan
KE	Bonobo	Lola Ya Bonobo	25.0	Adult	Adult	Male	Orphan
KI	Bonobo	Lola Ya Bonobo	8.0	Adult	Subadult	Female	Orphan
LI	Bonobo	Lola Ya Bonobo	8.0	Adult	Subadult	Female	Mo.-reared
LO	Bonobo	Lola Ya Bonobo	11.0	Adult	Subadult	Male	Orphan
LUB	Bonobo	Lola Ya Bonobo	6.0	Adult	Juvenile	Female	Orphan
MI	Bonobo	Lola Ya Bonobo	8.0	Adult	Subadult	Female	Orphan
MO	Bonobo	Lola Ya Bonobo	11.0	Adult	Subadult	Male	Mo.-reared
OP	Bonobo	Lola Ya Bonobo	23.0	Adult	Adult	Female	Orphan
SI	Bonobo	Lola Ya Bonobo	10.0	Adult	Subadult	Male	Orphan
TC	Bonobo	Lola Ya Bonobo	30.0	Adult	Adult	Female	Orphan
CO	Chimpanzees	La Vallée des Singes	22.0	Adult	Adult	Male	-
JO	Chimpanzees	La Vallée des Singes	23.0	Adult	Adult	Male	-
LL	Chimpanzees	La Vallée des Singes	9.0	Adult	Subadult	Female	-
RO	Chimpanzees	La Vallée des Singes	21.0	Adult	Adult	Male	-
WO	Chimpanzees	La Vallée des Singes	21.0	Adult	Adult	Male	-

S2 Table. Ethogram of signals (i.e., gestures, vocalizations, facial expressions) and GRBs after (1–6).

**Type**	**Description**
**Gestures**	
Bang cage	Hand(s) (or feet) brought into hard audible contact with the cage mesh (can be repetitive)
Bipedal swagger	Side to side of forward and back movement while standing/walking bipedal (rarely also quadrupedal)
Dangle	Subject hangs from arm(s) from the top of the cage/mesh, with feet shaking loosely
Grab experimenter	Hand(s) firmly closed over part of experimenter's body
Hit object	Hand(s) brought into short hard contact with the surface of an object
Hit object with object	Object brought into short hard contact with another object
Jump	While in bipedal position both feet leave ground simultaneously with horizontal displacement
Knock mesh/object	Back of hand/knuckles brought into short hard audible contact with cage mesh/object (can be repetitive)
Move hose	Hose is displaced in one direction, contact with hose is maintained throughout
Present body	Body part moved to deliberately expose an area to experimenter's attention
Present genitals	Genitals moved to deliberately expose them to experimenter's attention
Reach	Arm is extended to the experimenter with hand in open, palm exposed position
Shake experimenter	Hand(s) is firmly closed over part of experimenter's body with repeated back and forth motion of hand
Shake head	Head moved back and forth in small, repeated side motion
Shake hose	Hand(s) firmly closed over hose with repeated up and down or side movements
Shake hand	Small repeated back and forth motion of hand(s) from wrist
Stomp	Sole of the foot/feet brought into short hard audible contact with surface
Touch experimenter	Palm of hand and/or body is brought to light contact with part of experimenter's body, for under 2 s. Also counted if subject attempts to touch experimenter through the mesh but fingers are too short to reach experimenter's body
Touch hose	Palm of hand and/or body is brought to light contact with hose, for under 2 s
**GRBs**	
Drop hose out of cage	Hose pushed through the mesh outside of cage and out of reach (from subject)
Hand back hose	One end of hose pushed through the cage mesh outside of cage, while hand is firmly closed over other end of hose
Prompt	Body/hand is moved in rapid, short, and tense movements, while hand is firmly closed over hose and while looking at experimenter
Simulate game action	Hose pushed back and forth through the mesh (mimicking the game's pull and relax movements)
Touch with hose	Hose pushed through the cage mesh outside of cage and brought to light contact over part of experimenter's body

**Vocalizations**	
Laughter	Voiceless breathing sounding like low-pitched grunting
Hoo	Quiet, relatively inconspicuous vocalization sounding like «*hoo*», mostly emitted as a single call
Peep	High-frequency, often closed-mouth vocalization; short in duration and flat, simple acoustic structure
**Facial expressions**	
Pout face	Lips pursed forward and curled outward in front (circular opening); lips pressed together at mouth corners
Tightened lips	Lips horizontally tensed; drawn inward or slightly baring teeth
Play face	Mouth opened with lips either in relaxed position covering the upper teeth completely and the lower teeth partially, or retracted, showing both upper and lower frontal teeth

S3 Table. Count of signals and game-related behaviors (GRB) used to reengage the partner across groups.

**Reengagement type**	**Signal/GRB type**	**Infant bonobos**	**Adult bonobos**	**Adult chimpanzees**
Gesture	Bang cage	0	0	1
Gesture	Bipedal swagger	0	0	2
Gesture	Dangle	2	0	0
Gesture	Grab experimenter	5	0	0
Gesture	Hit object	1	0	0
Gesture	Hit object with object	1	4	1
Gesture	Jump	1	0	0
Gesture	Knock mesh	0	0	2
Gesture	Move hose	3	0	0
Gesture	Present body	5	1	1
Gesture	Present genitals	1	2	0
Gesture	Reach	4	0	4
Gesture	Shake experimenter	2	0	0
Gesture	Shake hand	0	2	0
Gesture	Shake head	1	1	1
Gesture	Shake hose	3	8	5
Gesture	Stomp	0	0	3
Gesture	Touch experimenter	3	0	1
Gesture	Touch hose	0	1	2
GRB	Hand back hose	1	52	13
GRB	Drop hose out of cage	0	6	7
GRB	Prompting	0	5	6
GRB	Simulate game action	0	3	10
GRB	Touch experimenter with hose	1	2	9
Facial expression	Play face	2	3	0
Facial expression	Pout face	0	2	1
Facial expression	Tightened lips	0	0	1
Vocalization	Hoo	0	0	1
Vocalization	Laughter	0	1	0
Vocalization	Peep	0	1	0
