# Supplementary material for: Correction: Potential evidence of reengagement attempts following interruptions of a triadic social game in bonobos and chimpanzees
Source: PLoS One. 2025 Dec 12;20(12):e0338981. doi: 10.1371/journal.pone.0338981 (PMC12700399; doi:10.1371/journal.pone.0338981)
Supplement: S1 File — (PDF) [file pone.0338981.s001.pdf]

PLOS ONE

RESEARCH ARTICLE

Potential evidence of reengagement attempts following interruptions of a triadic social game in bonobos and chimpanzees

Raphaela Heesen[1*], Adrian Bangerter[2], Klaus Zuberbühler[3,4], Katia Iglesias[5], Federico Rossano[6], Jean-Pascal Guéry[7], Emilie Genty[2]

1 Department of Psychology, University of Durham, Durham, United Kingdom, **2** Institute of Work and Organizational Psychology, University of Neuchâtel, Neuchâtel, Switzerland, **3** Department of Comparative Cognition, Institute of Biology, University of Neuchâtel, Neuchâtel, Switzerland, **4** School of Psychology and Neuroscience, University of St Andrews, St Andrews, United Kingdom, **5** School of Health Sciences (HEdS-FR), HES-SO University of Applied Sciences and Arts of Western Switzerland, Delémont, Switzerland **6** Department of Cognitive Science, University of California San Diego, California, United States of America, **7** Zoological Parc La Vallée Des Singes, Vienne, France

* raphaela.m.heesen@durham.ac.uk

OPEN ACCESS

Citation: Heesen R, Bangerter A, Zuberbühler K, Iglesias K, Rossano F, Guéry J-P, et al. (2025) Potential evidence of reengagement attempts following interruptions of a triadic social game in bonobos and chimpanzees. PLoS ONE 20(3): e0292984. https://doi.org/10.1371/journal.pone.0292984

Editor: Guangyu Zeng, The Chinese University of Hong Kong, Shenzhen, CHINA

Received: October 2, 2023

Accepted: February 10, 2025

Published: March 26, 2025

Copyright: © 2025 Heesen et al. This is an open access article distributed under the terms of the Creative Commons Attribution License, which permits unrestricted use, distribution, and reproduction in any medium, provided the original author and source are credited.

Data availability statement: The datasets of this study can be found on figshare.com: https://doi.org/10.6084/m9.figshare.21175129.v13

Funding: The present research was supported by the Swiss National Science Foundation (Grant No. CR31I3_166331 awarded to AB and KZ). RH thanks Kölner Gymnasial- und

Abstract

When humans engage in joint action, they seem to so with an underlying sense of joint commitment, a feeling of mutual obligation towards their partner and a shared goal. Whether our closest living relatives, bonobos and chimpanzees, experience and understand joint commitment in the same way is subject to debate. Crucial evidence concerns how participants respond to interruptions of joint actions, particularly if they protest or attempt to reengage their reluctant or distracted partners. During dyadic interactions, bonobos and chimpanzees exhibit evidence of reengagement following interruptions of naturalistic joint activities with conspecifics, according to recent studies. Yet, data are still inconsistent for triadic games, where two social partners engage with each other socially by focusing on a common object. We addressed this issue by engaging $N = 23$ apes (5 adult chimpanzees, 5 infant bonobos, 13 adult bonobos) in a "tug-of-war" game with a human experimenter who abruptly stopped playing. Following interruptions, adult apes readily produced communicative signals towards the experimenter (>60% of subjects on first trial), which we interpreted as reengagement attempts of their passive social partner, with no group differences in this respect. Infant bonobos, by contrast, communicated rarely with the experimenters compared to adult bonobos, and never during their first trial. Crucially, when infant bonobos signaled to passive partners, they predominantly used tactile signals, but rarely exhibited behaviors related to the game, which were instead commonly seen in adults. It is thus possible that bonobos and chimpanzees share some of the basic motivational foundations for joint commitment, yet that this capacity is subject to developmental effects.

Introduction

Many social animals engage in collaborative activities where two or more participants work towards a goal that would be unreachable by individuals. For instance, chimpanzees, whales,

Stiftungsfonds and EG thanks the Service de l'Egalité des Chances of the University of Neuchâtel for supporting their field stay in DRC. The funders had no role in study design, data collection and analysis, decision to publish, or preparation of the manuscript.

Competing interests: The authors have declared that no competing interests exist.

hyenas, and fish engage in group hunting [1–4], ants cooperatively transport objects [5,6], and meerkats mob predators [7]. Humans, though, are claimed to engage in *joint actions* with a presumably different underlying psychology that enables them to 'share' their intentions [8,9]. Shared intentionality may form the basis of many cultural achievements, social institutions, and language [10]. This quality also appears to encompass the formation of *joint commitment* – a concept that describes a feeling of mutual obligation towards the partner to bring a joint action to completion, and which supposedly underpins all human collaborative activities [9,11–13].

Empirically, joint commitment appears to manifest in specific behavioural patterns [14,15], although there is some debate on whether behavioural markers can truly represent mental constructs as such [16]. Possible behavioural correlates of joint commitment include ostensive signalling upon entering, maintaining, and exiting joint activities [14,15,17–21] and, when faced with interruptions, attempts to reinstate interactions in a coordinated fashion [22–24]. Humans in some cultural settings perceive sudden, not mutually ratified, interruptions as socially inappropriate [19,25], which typically leads to negative emotional reactions and corrective actions towards the partner [26–29], though this evidence mainly stems from observations and experiments conducted in Western, Educated, Industrial, Rich and Democratic (WEIRD) populations.

Interruptions of joint activities nonetheless provide a (currently the most applied) means to comparatively investigate joint commitment. One former paradigm consists of an experimenter abruptly disengaging from triadic games involving objects, such as to bounce a wooded block on a trampoline by holding it on opposite sites [26,27,30]. Moreover, Gräfenhain and colleagues [26] tested two- to three-year-old children's reactions to an interruption initiated by an experimenter in either a no-commitment condition (i.e., child and experimenter each play on their own) or a joint commitment condition (i.e., they play the game together). Three-year-olds, more than two-year-olds, attempted to reengage partners significantly more often in the joint- than the no commitment condition, suggesting a developmental trajectory of joint commitment. Young children thus already appear to understand that dissolving a commitment requires mutual agreement [28]. The understanding of joint commitment seems to emerge gradually, starting around the age of three and becoming even more profound at the age of five years, alongside a more general awareness of shared intentions and social norms [31].

On the other hand, investigations of whether non-human animals, notably our closest relatives, the great apes, can experience something akin to joint commitment have led to inconclusive results. While some researchers argue that joint commitment is human-unique [10,30], contrasting evidence on how great apes coordinate joint actions with peers, both naturally and in experimental situations, have reopened discussions [20,24,32–37]. Despite this evidence, joint commitment continues to be claimed a uniquely human capacity, primarily because of one influential comparative study on human children and three young chimpanzees, aged between 33 and 51 months [30]. Here, the children, but none of the chimpanzees, behaved and communicated in ways that suggests attempts to reengage the reluctant human experimenters, which was taken as evidence of joint commitment in humans yet not in apes. Reengagement in human children was interpreted as an attempt to repair with others the breakdown of a joint "we" [31]. However, subsequent studies testing older apes between 3-7 years and in less complex social interactions came to different conclusions, both concerning interactions with human experimenters [34,36,38] as well as joint activities with conspecifics [24,32,37]. These findings suggest that age may be a determining factor on whether (or not) apes reengage partners after interruptions. Moreover, considering newer reports on reengagement in bonobos [24,36], species differences in terms of reengagement behaviour could

equally explain negative findings in chimpanzees [30]. Potential variation between bonobos and chimpanzees might be explained by different social structures: Bonobos also have a reputation of being less despotic and more tolerant than chimpanzees [39–41], though this pattern varies between groups and settings [e.g., [42]].

In this study, we contributed to the growing body of literature on the spontaneous reengagement of social partners following disruptions in joint activities [20,24,32,34,36]. We used a triadic game paradigm to look for evidence of reengagement attempts through use of communicative signals [as a potential behavioural correlate of joint commitment, as suggested by 26,30] in chimpanzees and bonobos, and different age classes in bonobos. Specifically, we implemented a "tug of war" game between human experimenters and ape subjects. The game started by pulling a garden hose back and forth, and despite the competitive pulling aspect, the apes needed to act cooperatively by self-handicapping their pulling strength to accommodate their comparatively less powerful human partner, ensuring the game continues. After a few iterations, the experimenter then suddenly stopped by letting go of the hose. Following previous work [26,30], we predicted that if apes had some form or sense of joint commitment, they would feel committed to the shared activity and thus attempt to reengage the human experimenter when the game was interrupted, for instance by using game-related behaviours (hereafter: GRBs, including for instance the handing back of the object) and communicative signals.

To address potential age effects on reengagement behaviour, we compared infant and adult bonobos, expecting infants to be less likely to reengage than adults given fewer experiences in coordinating joint activities, notably with objects. We further manipulated the experimenter's attentional state, by either looking towards ("still-faced condition") or away from the subject ("back-turned condition"), as an additional source of variation in reengagement behaviour. We expected subjects to be more likely to attempt resuming the game when experimenters looked at the subject, compared to when being turned away, as gaze might be interpreted as a signal of availability, or readiness for interaction. This assumption is based on research in apes and human children, which showed that gaze represents a potential coordination device (at least under relaxed, affiliative and playful conditions), with eye contact being understood as a signal to engage [20,43,44].

Materials and methods

Ethics statement

We received ethical agreement for this study from the Commission d'Ethique de la Recherche of the University of Neuchâtel (agreement number: 01-FS-2017), the internal ethical committee of La Vallée des Singes, and from the Ministère de la Recherche Scientifique et Technologie de la République Démocratique du Congo (research permit number: MIN.RST/SG/180/020/2018). The individual pictured in Fig 1b (RH) has given written informed consent (as outlined in PLOS consent form) to publish these case details.

Study groups

Infant bonobos. Data on five infant bonobos (*Pan paniscus*, mean age = 3.3 y; SD = 0.9 y; range = 2.5-4.5 y; 2 females; 3 males, see S1 Table) were collected from October 2018 until December 2018, at the Lola Ya Bonobo sanctuary in the Democratic Republic of Congo. The infants were orphans, mostly victims of the bushmeat trade and confiscated by authorities. They had been cared for by humans from the moment of their arrival at the sanctuary. Each infant was cared for by a human surrogate mother for a few years before being introduced into an existing social group. During the day, they could range freely in an outdoor enclosure

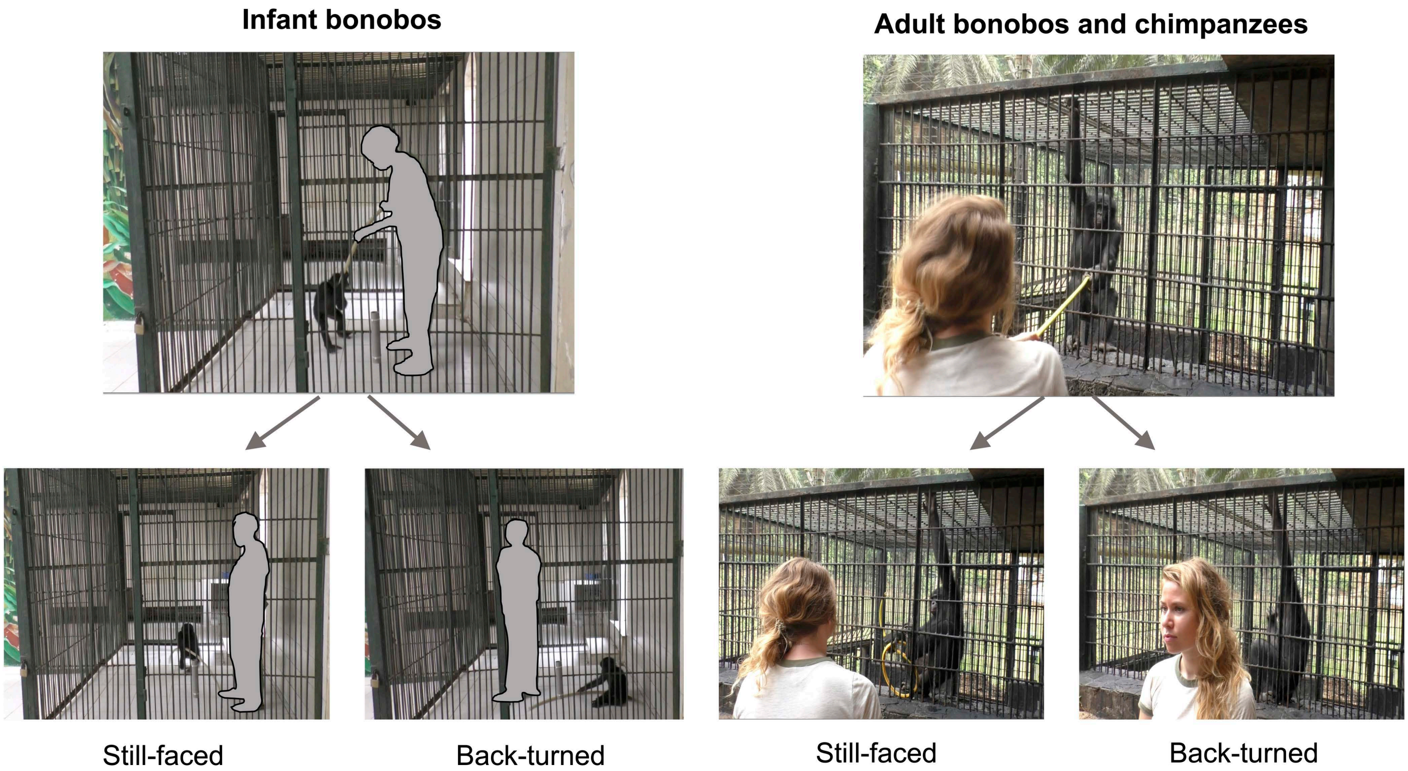

Fig 1. Experimental set-up for infant bonobos (a) and for adult bonobos and chimpanzees (b). When testing infant bonobos, the experimenter remains inside of the cage and engages in a tug-of-war game with the subject in the cage, using a garden hose. When testing adults, the experimenter stands outside the cage and engages in a tug-of-war game with the subject through the cage mesh, also using a garden hose. In the still-faced condition, the experimenter interrupts the game and faces the subject while remaining still; in the back-turned condition, the experimenter interrupts the game and turns their back to the subject while remaining still. The experimenter in the left plot is masked as we do not have their consent to publish their image.

https://doi.org/10.1371/journal.pone.0292984.g001

of approximately 500 m^2, comprising a forested patch that offered climbing opportunities. In addition, the enclosure was equipped with climbing structures, ropes, a pool, and a trampoline. The infants were free to interact with other orphans or the human surrogate mothers, who were always present in the enclosure. The infants were bottle-fed with a mixture of cow milk and cereals with water twice a day, and additionally received fruits, sugar cane, peanuts, and vegetables. Each individual received 5 l of water per day. On rainy days, an indoor enclosure was available (approximately 150 m^2), provided with climbing structures and ropes and the testing isolation cage (5 m^2). At night they slept in the indoor enclosures (each cage sized about 2 m^2; 2 infants per cage), which were furnished with hammocks.

Adult bonobos. Our experiment included thirteen subadult and adult bonobos, hereafter referred to as "adult bonobos" (mean age = 13.5 y; SD = 8.2 y; age range: 6.0 - 30.0 y; 9 females; 4 males; see S1 Table), also housed at the Lola Ya Bonobo sanctuary (i.e., data also collected from October 2018 until December 2018). At the time, the bonobos lived in three different social groups: Group 1: 20 individuals, including 11 females; Group 2: 19 individuals, including 8 females; Group 3: 15 individuals, including 8 females. The groups inhabited three 8-15 ha enclosures, consisting of streams, swamps, lakes, primary rainforest, and grassy open areas. The enclosures were separated by fence. They were provided with food by caregivers in four feedings per day (6-9 types of vegetables per day at 9 am, 2-4 types of fruits per day at 11 am and again at 4 pm, and protein balls at 2 pm), but could also range freely within their enclosures to forage for herbaceous vegetation and wild fruits. Water was available from

streams, lakes, and keepers (provided through bottles during the day). During the night, the groups were held in 75 m^2 dormitories furnished with hammocks.

Adult chimpanzees. At the time of study, the chimpanzee group consisted of seven chimpanzees (*Pan troglodytes verus)*, of which two individuals did not participate in the study (two females, aged 10 and 11 years). Thus, we tested five adult chimpanzees (mean age = 19.2 y; SD = 5.8 y; range: 9.0 - 23.0 y; 1 female; 4 males; see S1 Table), housed at the zoological park of La Vallée des Singes, France. The chimpanzees were tested between May and October 2018. The chimpanzee facility included an outside enclosure with a large forest area and climbing structures in grassy areas (7,500 m^2), and an inside enclosure with enrichment and various climbing structures (220 m^2). In stable weather conditions, the group was kept outside. Food was distributed five to six times a day and includes daily rations of primate pellets, fruits, vegetables, and rice. The chimpanzees were also occasionally fed with nuts, meat once a week, and eggs twice a week, and can forage for natural vegetation in their outdoor enclosure (wild berries, herbaceous vegetation). Fresh water was always available from a source at the building and a stream surrounding the island.

Experimental design and procedure

We tested subjects using a tug-of-war game in which a human experimenter and an ape pulled on opposite ends of a plastic garden hose through the mesh or bars of their indoor or outdoor enclosures (adult bonobos and chimpanzees; Fig 1b), and from within the inside of the testing cage (infant bonobos; Fig 1a). For the game to work, i.e., to obtain repeated sequences of back-and-forth pulling movements of the hose, both partners had to alternate pulling the hose, and the apes (who are significantly stronger than humans, at least from a subadult age) needed to adjust their pulling force; otherwise, the hose would be pulled into the cage, causing the game to end. The chimpanzees had been in contact with garden hoses prior to the experiment, either as part of the cleaning equipment of keepers or as a toy inside the cage. For the bonobos, it was uncertain whether they had any experience with garden hoses. Thus, we implemented a habituation period by distributing 2-3 garden hoses identical to those used in the experiment in all enclosures one week before the start of the experiment. Participation was voluntary, meaning that during testing no adult subject was isolated from the rest of their social group and they could come or leave as desired. Infant bonobos on the other hand were isolated from the other orphans during testing to avoid regular disturbances by other playful infants. Yet, during testing, the orphans' surrogate human mother was always present in the cage for emotional support.

All sessions were filmed from outside the cage using a Panasonic HC-V770 Camcorder on a tripod, equipped with an external Sennheiser microphone (MKE 400). Trials began either by an ape subject handing the hose to the experimenter or by the experimenter handing the hose to the subject, accompanied by verbal encouragement. As soon as both held the hose, the goal was to establish a sequence of rapid back-and-forth pulling movements ("tug-of-war"). This movement was then suddenly interrupted by the experimenter who dropped the hose (for interruption period durations, see details below). During an interruption, the experimenter stood still, either facing the subject (still-faced condition: condition 1) or turning their back to the subject (back-turned condition: condition 2), see Fig 1. Two additional conditions were included in the original study plan but could not be implemented in a sufficiently consistent fashion across groups to be included in the study; we also could not reach enough trials across groups to gather a sufficient sample for analysis. These two failed conditions, described in further detail in S1 Text, included a "clumsy experimenter" and "third party interruption" condition. The first involved an attempt to either simulate a naive experimenter who did not

know how to play the game. The second implied that, during the ongoing game between ape and human experimenter, a secondary human experimenter distracted the first experimenter by engaging them in a conversation, thus being responsible for causing the interruption.

Trials were applied opportunistically given that the subjects' individual motivation to participate in the game varied across testing days. Consequently, a daily experimental session (total sessions conducted = 69; mean = 3 per subject) could include one or more trials. Each of the 4 initial conditions (still-faced and back-turned, as well as the two failed conditions, see S1 Text) were presented in a randomized order, and could be administered once or several times depending on the subject's motivation. As much as possible, we tried to counterbalance the order of conditions across testing days. We tried to test all subjects at least once in each condition, but participation depended largely on their motivation. Hence, not every individual could be tested in each condition if they decided to not return to the game.

Infant bonobos. Infant bonobos were tested in the isolation cage of their indoor enclosure (Fig 1a). Experimenters were the infants' human surrogate mothers, to whom they are emotionally attached (i.e., except for 8 trials, which were conducted by RH). The subjects were carried to the testing compartment by their surrogate mothers who entered the cage with them. The hose was placed in the cage before they arrived. Once they entered the testing cage, the door was locked during the testing. Each testing session was stopped after about 15 min even if no trial had been completed. If the infants were motivated to play, the session could be longer. For each session and subject, experimenters were selected depending on their availabilities during the day. Thus, the order in which infants were tested was based on the surrogate mother's most convenient schedule. On a testing day, subjects were called into the testing area and brought in by their surrogate mother. If they were not motivated, they were called in again later, or testing was postponed to another day. Following a previous experiment with bonobos using a similar paradigm [34], the interruption periods lasted 30 s. We analysed all behaviours and communicative signals occurring during this period. Surrogate mothers had reported no prior experience in conducting the tug-of-war game with infant bonobos. In total, we conducted 29 trials with infant bonobos (still-faced: $N = 17$; *median* [*IQR*] = 3 [2; 4] trials per individual; back-turned: $N = 12$; *median* [*IQR*] = 2 [2; 3] trials per individual).

Adult bonobos. The subjects were tested in their indoor and outdoor facility, wherever the experimenter and the subjects could play the game through the cage mesh. For group 3 and 1, this was primarily in their isolation cage or indoors, e.g., Fig 1b. For group 2, this was outside either in the isolation cage or through the mesh doors of the outdoor enclosure. Four different persons acted as experimenters: two women (authors RH and EG, who were unfamiliar to the bonobos) and two men (zookeepers who were familiar to the bonobos, working full-time with them). Like for infant bonobos, the interruption periods also lasted 30 s, during which we investigated all behaviours and communicative signals. The session lasted as long as the bonobos were motivated to participate. For adult bonobos, no session cap of 15 min was applied; the subjects were not isolated from the rest of their group like infant bonobos but could come and go as they pleased (i.e., in contrast to the infant bonobos, the testing did not restrict the adult subjects' abilities to engage in other daily social activities). All testing was voluntary, and participants were recruited based on their motivation to play with the experimenter. Keepers and researchers had reported no prior experience in conducting the tug-of-war game with the adult bonobos. The only exception is one mother-reared individual (Moyi, "MO", S1 Table) who had engaged in a tug-of-war like game with a cotton rag with author EG in July 2013, an event that initially sparked the idea to use such a game for this experiment. We conducted 52 trials with adult bonobos (still-faced: $N = 30$; *median* [*IQR*] = 1 [1; 4] trials per individual; back-turned: $N = 22$; *median* [*IQR*] = 1 [1; 2.5] trials per individual).

Adult chimpanzees. The game was played inside the holding building, and through the cage mesh, at a location wherever subjects spontaneously engaged in the game, but always in the indoor enclosures either in the mornings or evenings. Five persons acted as experimenters. These included two women (author RH who interacted rarely with the chimpanzees and a zookeeper who worked approx. two times a month with the chimpanzees) and three men (two zookeepers working approx. five to ten times a month with the chimpanzees, and another who worked full-time with chimpanzees). The keepers' participation in a testing session was determined based on their availabilities on a given testing day. To allow comparison with chimpanzees in the study by Warneken et al. [30], the interruption periods were aimed at lasting 15 s. However, in some cases the keepers' reactions were slightly delayed, resulting in interruption periods that varied in length (lasting on average 22.1 s, $SD = 7.76$ s). To allow for a more consistent evaluation of all behaviors and communicative signals between trials, we therefore only analyzed behaviors or communicative signals occurring during the first 15 s of the interruption period. As for the other groups, the session lasted as long as the chimpanzees were motivated to participate in the game. Keepers and researchers had no prior experience in conducting the tug-of-war game with the chimpanzees. We were able to conduct 58 trials with adult chimpanzees (still-faced: $N = 27$; *median* [*IQR*] = 5 [3; 6] trials per individual; back-turned: $N = 31$; *median* [*IQR*] = 3 [3; 4] trials per individual).

Video coding

We coded game-related behaviors (GRBs) and signals (i.e., gestures, vocalizations, facial expressions) deployed during interruption periods using the ELAN package, v 5.2 [45]. During interruption periods, we annotated whether subjects attempted to reengage their partner or not. A reengagement attempt was annotated (yes = 1/no = 0) if one or several GRBs or communicative signals were produced during an interruption (see Table 1 and ethogram in S2 Table).

Coding reliability. We carried out an inter-observer reliability test between two great ape communication researchers (authors RH and EG). The test compared the coding on the presence or absence of GRBs and signals (i.e., which indicated the presence or absence of reengagement attempts), based on the ethogram in S2 Table, occurring during the interruption periods in both the still-faced and back-turned conditions. Inter-rater coding was done for a total of 22 trials, representing 15% of trials for each group out of their total number of trials as reported in the main text. The result for signals was Cohen's $\kappa = 0.81$ (agreement 98.9%) and for GRB Cohen's $\kappa = 0.84$ (agreement 95.5%), indicating almost perfect agreement [47].

Table 1. Communicative behaviours indicating reengagement during interruption periods.

Reengagement types	Description
Game-related behaviours (GRB)	Subject encouraged the experimenter to reinstate the game by producing communicative behaviours with reference to the game object. This could include manipulating the game object, either by handing it back to the experimenter, prompting the experimenter (a jerky, rapid back-and-forth movement of the game object through the mesh simulating the game action), touching the experimenter with the object, or dropping it outside the cage, see S2 Table.
Signals	Subject produced communicative signals towards the experimenter, including vocalizations, gestures, and facial expressions, see S2 Table. Signal types were categorized based on previous research of great ape intentional communication and have no direct link to the game procedure/action itself [46]. Gestures used to merely get into bodily contact with the experimenter (i.e., infants embracing their surrogate mothers for contact) without additional reengagement cues were not counted as reengagement signals.

https://doi.org/10.1371/journal.pone.0292984.t001

Statistical analysis

We present the results for each group separately on the number of trials during which subjects attempted to reengage experimenters, as well as in which subjects used GRBs/signals. These results are presented both for first trials only and across all trials, providing a clearer view of behaviors when the task was novel and after subjects had the opportunity to habituate to it, particularly for those subjects who participated in many trials compared to those who engaged in only a few (see Table 3).

As subsequent analysis, we considered variation in reengagement attempts, GRBs and signal percentages based on differences in the number of trials administered across subjects. We computed percentages of reengagement attempts by dividing each participant's number of reengagement attempts by its number of completed trials multiplied by 100. To describe the results, we presented median percentage of these reengagement attempts, as well as the interquartile range (IQR), i.e., first and third quartile, considering the full range of data (including endpoints) and applying linear interpolation ("=QUARTILE.INC()" standard function in Excel v 16.92). Likewise, for signals and GRBs, we computed the percentage of reengagement attempts in which subjects used signals and GRBs by dividing the number of reengagements attempts *with* signal or GRBs by the number of subjects' total of *reengaged* trials multiplied by 100; in our results, we presented the median of these percentages along with the IQRs.

We further tested the effect of condition [still-faced ("condition 1") and back-turned ("condition 2") *within* group], age (comparison infants and adult bonobos) and group (comparison adult chimpanzees and adult bonobos) on the percentages by which individuals produced a) reengagement attempts, b) GRBs, and c) signals as per reengaged trials. We used non-parametric Wilcoxon signed-rank tests for within-group comparisons and Mann-Whitney U tests (i.e., Wilcoxon's rank-sum test) for between-group comparisons in R Studio (v 2023.12.1+402). Following [48], we report results for the Wilcoxon signed-rank tests via *p*-values, and results for the Mann-Whitney U tests via the test statistic provided in R (W) as well as respective *p-value*s. To indicate the magnitude of existing effects, we additionally computed effect sizes ("*r*") for between-group comparisons that were significant at $alpha = 0.05$ following the R function provided in [48]. Effects exceeding the threshold of 0.3 indicate medium effects (accounting for 9% of the total variance) and effects exceeding 0.5 indicate large effects (accounting for 25% of the variance) [48]. Subjects who only participated in one condition were excluded from the analysis of the Wilcoxon signed-rank tests. Whenever sample sizes were too small (e.g., comparing GRB and signal use between groups and within condition), we reported the combined results for both conditions.

To assess whether any inconsistencies or unwanted variation in the experimental design across groups could have affected our results, we decided to conduct additional post hoc analyses as described below.

Since chimpanzees' and bonobos' interruption periods differed in duration (i.e., 15 sec and 30 sec, respectively), we conducted a post hoc group comparison between adult bonobos and chimpanzees in which we only considered reengagement attempts within the first 15 s.

Moreover, our experiment was affected by subjects' level of motivation, insofar as each subject participated voluntarily without being forced or separated from their social group. This yielded an unwanted and large variation in the number of trials (thus experience in the game) across subjects, which may have affected our results on reengagement rates. To take this into account, we conducted a Spearman's rank correlation test to examine the relationship between number of trials and reengagement percentages.

Furthermore, to understand whether the experimenters' familiarity with the subjects influenced reengagement attempts, we tested whether there were differences in reengagement rates depending on the familiarity with the experimenter ("familiar" for keepers and surrogate

mothers; "unfamiliar" for researchers who rarely engaged with the apes outside of testing times).

Since some of our adult bonobo subjects are orphans and were raised by human surrogate mothers rather than by their natural mothers ($N = 10$; S1 Table), early experiences through human interactions could have enhanced reengagement behaviour. Therefore, the subjects of this group cannot be directly compared without additional verification of rearing impact. Thus, we provide additional results on reengagement separately for orphans and non-orphan adult bonobos.

Results and discussion

Descriptive results on latencies of reengagement responses across groups are presented in Table 2.

Infant bonobos

Do infant bonobos reengage passive partners?. On first trial, none of the infant bonobos reengaged the experimenters (0%, Table 3). When considering first and subsequent trials, four out of five infant bonobos reengaged the experimenter at least in one trial (80%, Table 3). When considering reengagement percentages based on all trials (Table 3), we found that in the still-faced condition, infant bonobos attempted to reengage experimenters in six out of seventeen trials (*median* [*IQR*] = 33.3% [25.0%; 42.9%]). In the back-turned condition, they attempted to reengage experimenters in four out of twelve trials (*median* [*IQR*] = 0.0% [0%; 50.0%]). There was no statistical difference in reengagement attempts between conditions ($p = 0.789$).

How do infant bonobos reengage passive partners?. On first trial, none of the infant bonobos used GRBs or signals (0%, Table 3). When considering first and subsequent trials, one out of five infant bonobos reengaged the experimenter using GRBs (20%) and four out of five infant bonobos did so producing signals in at least one trial (80%, Table 3).

Out of all reengaged trials ($N = 10$), we found that only one subject (Kwango, 2.5 y) produced GRBs (i.e., on two out of six reengaged trials). Due to low sample size, we were unable to statistically compare GRB use between conditions for infant bonobos who reengaged. By contrast, infant bonobos used signals in every reengaged trial ($N = 10$), yielding identical signal use across conditions. The signals ($N = 34$) produced by infant bonobos contained 94.1% gestures, 5.9% facial expressions and 0% vocalizations, see S3 Table for counts on signal types.

Adult bonobos

Do adult bonobos reengage passive partners?. On first trial, nine out of thirteen adult bonobos reengaged the experimenters (69.2%, Table 3). When considering first and subsequent trials, eleven out of thirteen adult bonobos reengaged the experimenter in at least one trial (84.6%, Table 3).When considering reengagement percentages based on all trials (Table 3), we found that in the still-faced condition, adult bonobos attempted to reengage experimenters in twenty out of thirty trials (*median* [*IQR*] = 66.7% [56.3%; 100%]). In the

Table 2. Response latencies (s) of reengagement attempts across groups.

Group	*Median* (s)	*IQR* (s)
Infant bonobos	10.3	5.6; 17.4
Adult bonobos	9.1	4.7; 18.2
Adult chimpanzees	4.5	3.0; 11.2

https://doi.org/10.1371/journal.pone.0292984.t002

Table 3. Reengagement, GRB and signal use as per subject and across groups (a: infant bonobos, b: adult bonobos, c: adult chimpanzees). Rearing status for bonobos indicated as ᵐ (mother-reared) and ᵒ (orphan). R = reengagement attempt; G = Use of game related behavior(s); S = Use of signal(s). Red color = Evidence for behavior in question absent; green color = Evidence for behavior in question present. Blank cells indicate lack of trials. Combines both conditions.

a) Infant bonobos

		Trial																																
Subjectrearing	Reengagement%	1			2			3			4			5			6			7			8			9			10			11		
		R	G	S	R	G	S	R	G	S	R	G	S	R	G	S	R	G	S	R	G	S	R	G	S	R	G	S	R	G	S	R	G	S
BA^{o}	14.3																																	
BI^{o}	50.0																																	
KW^{o}	54.5																																	
LA^{o}	25.0																																	
LU^{o}	0.0																																	

b) Adult bonobos

		Trial																																
Subjectrearing	Reengagement%	1			2			3			4			5			6			7			8			9			10			11		
		R	G	S	R	G	S	R	G	S	R	G	S	R	G	S	R	G	S	R	G	S	R	G	S	R	G	S	R	G	S	R	G	S
BO^{o}	50.0																																	
EL^{m}	50.0																																	
IS^{o}	100.0																																	
KE^{o}	55.6																																	
KI^{o}	50.0																																	
LI^{m}	100.0																																	
LO^{o}	60.0																																	
LUB^{o}	0.0																																	
MI^{o}	50.0																																	
MO^{m}	50.0																																	
OP^{o}	50.0																																	
SI^{o}	0.0																																	
TC^{o}	100.0																																	
		12			13			14			15																							
		R	G	S	R	G	S	R	G	S	R	G	S																					
BO^{o}	50.0																																	
EL^{m}	50.0																																	
IS^{o}	100.0																																	
KE^{o}	55.6																																	
KI^{o}	50.0																																	
LI^{m}	100.0																																	
LO^{o}	60.0																																	
LUB^{o}	0.0																																	
MI^{o}	50.0																																	
MO^{m}	50.0																																	
OP^{o}	50.0																																	
SI^{o}	0.0																																	
TC^{o}	100.0																																	

c) Adult chimpanzees

		Trial																																
Subject	Reengagement%	1			2			3			4			5			6			7			8			9			10			11		
		R	G	S	R	G	S	R	G	S	R	G	S	R	G	S	R	G	S	R	G	S	R	G	S	R	G	S	R	G	S	R	G	S
CO	77.8																																	

(Continued)

Table 3. (Continued)

a) Infant bonobos

		**Trial**																																
**Subjectrearing**	**Reengagement%**	**1**			**2**			**3**			**4**			**5**			**6**			**7**			**8**			**9**			**10**			**11**		
		**R**	**G**	**S**	**R**	**G**	**S**	**R**	**G**	**S**	**R**	**G**	**S**	**R**	**G**	**S**	**R**	**G**	**S**	**R**	**G**	**S**	**R**	**G**	**S**	**R**	**G**	**S**	**R**	**G**	**S**	**R**	**G**	**S**
JO	81.3																																	
LL	100.0																																	
RO	66.7																																	
WO	33.3																																	
		**12**			**13**			**14**			**15**			**16**			**17**			**18**			**19**			**20**			**21**			**22**		
		**R**	**G**	**S**	**R**	**G**	**S**	**R**	**G**	**S**	**R**	**G**	**S**	**R**	**G**	**S**	**R**	**G**	**S**	**R**	**G**	**S**	**R**	**G**	**S**	**R**	**G**	**S**	**R**	**G**	**S**	**R**	**G**	**S**
CO	77.8																																	
JO	81.3																																	
LL	100.0																																	
RO	66.7																																	
WO	33.3																																	
		**23**			**24**			**25**			**26**			**27**			**28**			**29**			**30**			**31**			**32**					
		**R**	**G**	**S**	**R**	**G**	**S**	**R**	**G**	**S**	**R**	**G**	**S**	**R**	**G**	**S**	**R**	**G**	**S**	**R**	**G**	**S**	**R**	**G**	**S**	**R**	**G**	**S**	**R**	**G**	**S**			
CO	77.8																																	
JO	81.3																																	
LL	100.0																																	
RO	66.7																																	
WO	33.3																																	

https://doi.org/10.1371/journal.pone.0292984.t003

back-turned condition, they attempted to reengage experimenters in nine out of twenty-two trials (*median* [*IQR*] = 33.3% [0%; 100%]). There was, however, no statistical difference in reengagement attempts between conditions ($p = 0.202$).

How do adult bonobos reengage passive partners?. On first trial, nine out of thirteen adult bonobos used GRB (69.2%) and only two out of thirteen subjects used signals (15.4%), see Table 3. When considering first and subsequent trials, eleven out of thirteen adult bonobos used GRBs in at least one of their trials (84.6%), while only six out of thirteen subjects produced signals in at least one of their trials (46.2%, Table 3).

Out of all reengaged trials ($N = 29$), the adult bonobos used GRB in twenty-eight trials (*median* [*IQR*] = 100% [100%; 100%]). By contrast, they produced signals only in thirteen out of twenty-nine reengaged trials (*median* [*IQR*] = 20% [0%; 65.3%]). The signals ($N = 26$) consisted of 73.1% gestures, 19.2% facial expressions and 7.7% vocalizations, see S3 Table for counts on signal types. Between conditions, there was no statistical difference in the way bonobos produced GRBs ($p = 1$; still-faced: *median* [*IQR*] = 100% [100%; 100%], $N = 20$ reengaged trials; back-turned: *median* [*IQR*] = 100% [100%; 100%], $N = 9$ reengaged trials). Although there were fewer signals produced in the back-turned condition compared to the still-faced condition, this result did not reach statistical significance, possibly due to low sample size ($p = 1$; still-faced: *median* [*IQR*] = 75.0% [0%; 100%], $N = 20$ reengaged trials; back-turned: *median* [*IQR*] = 0% [0%; 18.75%], $N = 9$ reengaged trials).

Adult chimpanzees

Do adult chimpanzees reengage passive partners?. On the first trial, three out of five adult chimpanzees reengaged the experimenters (60%, Table 3). When considering first and

subsequent trials, all five chimpanzees reengaged the experimenters on at least one of their trials (100%, Table 3).

When considering reengagement percentages based on all trials (Table 3), we found that in the still-faced condition, adult chimpanzees attempted to reengage experimenters in twenty-one out of twenty-seven trials (*median* [*IQR*] = 83.3% [66.7%; 91.7%]). In the back-turned condition, they attempted to reengage experimenters in twenty-two out of thirty-one trials (*median* [*IQR*] = 75.0% [66.7%; 100%]). There was no statistical difference in reengagement attempts between conditions ($p = 0.584$).

How do adult chimpanzees reengage passive partners?. On first trial, two out of five adult chimpanzees used GRB (40%) and only one out of five used signals (20%), see Table 3. When considering first and subsequent trials, all adult chimpanzees used GRBs in at least one of their trials (100%), while four out of five produced signals in at least one of their trials (80%, Table 3).

Out of all reengaged trials ($N = 43$), we found that adult chimpanzees used GRB in thirty trials (*median* [*IQR*] = 100% [61.5%; 100%]). By contrast, they produced signals only in twenty out of forty-three reengaged trials (*median* [*IQR*] = 50% [33.3%; 50.0%]). The signals ($N = 26$) consisted of 88.5% gestures, 7.7% facial expressions and 3.8% vocalizations, see S2 Table for signal type counts. Between conditions, there was no difference in the way chimpanzees produced GRBs ($p = 0.371$; still-faced: *median* [*IQR*] = 100% [54.5%; 100%], $N = 21$ reengaged trials; back-turned: *median* [*IQR*] = 100% [91.7%; 100%], $N = 22$ reengaged trials). Although there were fewer signals produced in the back-turned condition compared to the still-faced condition, this difference did not reach statistical significance ($p = 0.181$; still-faced: *median* [*IQR*] = 50.0% [50.0%; 60.0%], $N = 21$ reengaged trials; back-turned: *median* [*IQR*] = 32.5% [18.8%; 42.5%], $N = 22$ reengaged trials).

Do infant bonobos and adult bonobos differ in terms of reengagement attempts, use of GRBs, or signals?

In the still-faced condition, infant bonobos attempted to reengage experimenters significantly less often compared to adult bonobos ($W = 45.5$, $p < 0.05$, $r = -0.50$; Table 3). In the back-turned condition, infant bonobos attempted to reengage the experimenters in fewer trials compared to adult bonobos (Table 3), yet this difference was not statistically significant ($W = 34.5$, $p = 0.428$). In terms of reengagement behaviours, infants used significantly less GRBs across conditions compared to adults in reengaged trials ($W = 44.0$, $p < 0.001$, $r = -0.87$; Table 3). By contrast, adult bonobos were significantly less likely to use signals across conditions compared to infant bonobos in reengaged trials ($W = 4.0$, $p < 0.05$, $r = -0.62$; Table 3).

Do adult bonobos and adult chimpanzees differ in terms of reengagement attempts, use of GRBs, or signals?

There were no significant differences in reengagement attempts between adult bonobos and chimpanzees, neither in the still-faced condition ($W = 25$, $p = 0.817$) nor the back-turned condition ($W = 20.5$, $p = 0.437$). Furthermore, there were no significant differences between adult chimpanzees and bonobos in the use of GRBs ($W = 37$, $p = 0.135$) or signals ($W = 25.5$, $p = 0.861$) among reengaged trials.

Post hoc analyses

In line with the 30-sec analysis for bonobos, there were no significant differences in the way adult bonobos attempted to reengage experimenters compared to chimpanzees in 15 s interruption periods (still-faced condition: $W = 18$, $p = 0.303$; back-turned condition: $W = 16.5$, $p = 0.196$).

There also was no evident correlation between number of trials and reengagement percentages ($rho = 0.12$, $p = 0.58$), indicating no evidence of an increase in reengagement percentages for subjects with greater number of trials (thus, possibly more habituation of and experience with the task).

Likewise, we found no evidence that the familiarity of the experimenter had an impact on reengagement attempts ($W = 5.5$, $p = 0.389$).

Finally, when looking at rearing status in adult bonobos, we found that orphans' and non-orphans' reengagement percentage was nearly identical, suggesting no direct impact of rearing on reengagement behaviour (orphans $N = 10$: *median* [*IQR*] = 50.0% [50%; 58.9%]; mother-reared $N = 3$: *median* [*IQR*] = 50.0% [50.0%; 75%], see Table 3).

Discussion

Reengagement of passive social partners following an interruption of a joint action has previously been understood as a behavioural indicator of joint commitment [26,30]. Based on contradictory findings regarding reengagement behaviour in apes, our study was designed to expand previous research by assessing whether chimpanzees and bonobos reengage passive social partners in a novel triadic social game ("tug-of-war"). Our aim was to assess whether, by using a less complex but more species-adapted game (i.e., including some competitive element while retaining an overarching cooperative goal), and focusing on species-specific communicative signals, we could demonstrate evidence for reengagement behaviors in apes. With this paradigm, we intended to provide a more comprehensive study compared to former studies, which comprised very few and young chimpanzee subjects, and which mainly revealed negative findings on reengagement attempts in chimpanzees [e.g., 30]. Specifically, our aim was to examine whether the variation in findings across studies regarding apes' ability to reengage passive partners [e.g., 30,34,36] are affected by subject age, group, and the choice of the game. Our data suggested that adults, yet less so younger subjects, were motivated to engage in the game with human partners and to reinstate the game when interrupted using communication and GRBs. Before proceeding further with the discussion, we feel it is important to acknowledge that the behaviors identified as reengagement attempts should still be interpreted with caution, as they may qualify as general signaling directed toward humans. In other words, while the observed behaviors we here interpreted as reengagement attempts (following previous research on children and chimpanzees [30]), they could also reflect a broader intent to initiate interactions. To address this ambiguity, we recommend incorporating additional baseline controls in future experiments, such as a "social non-game" control condition, in which one could measure communicative behavior during still-face or back-turned interruptions that were not preceded by the tug-of-war game. Such a control condition would permit to compare signaling rates in contexts with and without a prior tug-of-war game, and it should be applied both in apes as well as in humans.

While we remain cautious in our interpretation, we nonetheless believe that the communicative behaviors and signals observed, particularly in adult apes, could qualify as reengagement behaviors. This is so because the behaviors and signals produced during interruption phases of the tug-of-war game frequently involved movements related to the game, often incorporating the game object itself, suggesting an understanding of the game and intentions to reengage. Although there were no differences in presumed reengagement rates between adult chimpanzees and bonobos, we found differences between infant and adult bonobos. Infant bonobos attempted to reengage partners much less frequently (and never on their first trial) compared to adult bonobos. Interestingly though, when considering all trials, four out of five infant bonobos reengaged the experimenter in at least one of their trials (Table 3),

suggesting the ability to reinstate a triadic game in principle. The major difference appeared to lie in the frequency of reengagement across the two age groups, with adult bonobos more readily reengaging compared to infant bonobos, as well as the *way* of reengaging passive partners: When attempting to reengage passive partners, adult bonobos used more GRBs than infants (irrespective of whether one considers behaviours on the first trial or all trials). Infants, by contrast to adults, relied mainly on tactile, gestural communication (without apparent relation to the game). Such a signalling pattern is supported by other studies examining signaling efforts across different age groups in apes, albeit unrelated to reengagement. They have highlighted developmental differences in signal use directed toward human experimenters. For example, younger chimpanzees (3–7 y) demonstrated fewer vocalizations, gestures, and instances of gaze alternation compared to older chimpanzees (8 + y) when communicating with a human experimenter about an inaccessible food reward [49]. This mirrors our results for infant bonobos.

The reported differences in our study suggest different levels of understanding of the joint nature of the game and potential roles of the social partners in adult- compared to infant bonobos. Given the use of signals - yet rarely GRBs - in infant bonobos, it is actually difficult to ascertain whether infants were attempting to reinstate the game after all. Instead, the infants' communication might be caused by fear of abandonment (i.e., caretaker turning away). This indicates a more profound understanding of the triadic game in adults as compared to infants; infant bonobos might not yet perceive the interaction as joint in the same degree as older individuals do, a skill that may be scaffolded with social experience and engagement in joint activities. Indeed, infant bonobos have a long development period and stay dependent on their mother until approximately 4-5 years of age [50]. It is possible that reengagement behaviour develops as individuals become more independent in terms of interactions with non-mother partners, gain interactive experience, as well as become more sensitive to social relationships [51,52]. Human children also become skilful at engaging in cooperative activities and reengaging passive partners following their third year of life [23,29,53,54]. Such developmental patterns might explain the negative findings of a former key study on this topic, where juvenile chimpanzees never attempted to reengage experimenters following interruptions [30].

One alternative explanation for the increased, especially tactile, gesture use in infant bonobos could be that infants were tested while the experimenter was in their cage, opposite to the adults, where the experimenter was standing outside the cage. If the adult subjects had had more opportunities to touch or grab the experimenter, they might have done so as well. Given the limits of our design, we cannot clarify this here; future research is needed to exclude this explanation. Our findings nonetheless suggest for the first time that in bonobos, ontogenetic differences could explain variation in reengagement behaviour and use of GRBs across groups. Future research with larger samples, more species, and ideally a longitudinal approach would be necessary to solidify the evidence on developmental trajectories of reengagement behaviour in apes.

A potential further factor to explain differences in reengagement rates between infant and adult bonobos could be discrepancies in rearing experiences across individuals. For instance, one study showed that early manifestations of cooperation varied across two groups of nursery-reared chimpanzees who experienced different caregiving styles in their first year of life [55]. Some of our bonobo subjects are orphans and were raised by human surrogate mothers rather than by their natural mothers (S1 Table). Such early-life experiences could have fostered reengagement behaviour since most of the orphans' early social experiences have been scaffolded by interactions with humans. However, the difference in rearing experience is unlikely to have had any impact on our results because none of the apes had any previous

experience in playing the tug-of-war games with humans. In support of this view, we found that adult bonobo orphans' and non-orphans' reengagement percentages were almost identical, suggesting no impact of human scaffolding on reengagement behaviour.

Yet also in humans the impact of the social environment could explain children's motivation to reengage passive partners. Research on the development of joint commitment in humans is entirely based on WEIRD samples [23,26–29], making it difficult to judge in how far empirical manifestations of this capacity are affected by culture and other social dimensions. For this reason, the generalizability of previous findings even in humans remains uncertain. Thus, given the limited evidence on joint commitment, and small sample sizes, human and ape researchers currently must remain cautious in generalizing their findings of some groups to the entire species. More evidence is needed including more samples with varying social and rearing conditions. More broadly, differences in developmental experiences influencing cognition and communication must be considered for both animals *and* humans alike [56].

Previous studies have examined reengagement behaviour mainly in younger individuals [30,34,36], but no consistent within-species comparison has yet been done to assess differences between younger and older age classes. Compared with previous studies, our findings contradicted those of [30], where chimpanzees made no attempts at reengaging a passive experimenter. Instead, our findings add to the growing evidence [24,32,34,36] that apes may possess some of the motivational preconditions to develop a basic understanding of joint commitment in both dyadic social interactions *and* triadic games. Critically, our current design implied leaving the object accessible to the subjects during interruption periods, which allowed for testing whether the apes would use the game object (garden hose) when attempting to reengage a partner, rather than keeping it to themselves. Indeed, the adult subjects not only demonstrated the motivation to engage in the shared triadic game through self-handicapping (i.e., by restraining physical strength when pulling the rope), they also frequently produced GRBs when reengaging a partner (e.g., handing back the hose when pulled inside the cage, simulating the game action, dropping the hose outside of the cage, see S2 and S3 Tables), constituting evidence of their intentions to resume the game as well as an understanding of how the game works. The apes' behaviours is comparable with the behaviours of human children when engaging in triadic games, when offering toys to- or reminding experimenters how the game was played [e.g., 26]. Although infant chimpanzees have not reengaged passive experimenters in a former study [30], infant bonobos in this study did so, albeit much less compared to adult subjects. Thus, our results show that, if conditions are right, even infant apes attempt to reengage experimenters in principle (albeit less so than adult bonobos and via less complex communicative means). Yet, given the lack of infant chimpanzees as comparison group, we cannot refute whether the difference between our infant bonobos and the infant chimpanzees tested by [30] could equally be related to species differences. To test this, future studies should apply this paradigm to reassess reengagement of interrupted triadic joint activities in several groups of infant chimpanzees.

Crucially, although the choice of differential interruption periods across groups (30s for bonobos and 15s for chimpanzees) was intentionally chosen to provide comparable data with previous studies, it represented a limitation for our group comparisons. An inspection of response latencies across groups (Table 2), however, showed that adult bonobos' response latencies were anyways longer than those of chimpanzees. Given this result, we believe that our choice of interruption periods naturally represents the reengagement response latencies of the two groups. The post hoc analysis further revealed stable results that when both groups are compared at 15 s.

Although our findings, along with previous research [24,32], support the hypothesis that adult bonobos and chimpanzees experience some basic form of joint commitment, these data do not yet provide conclusive evidence. For instance, apes may simply enjoy playing the tug-of-war game and understand that the game requires a cooperating human partner, without any sense of joint commitment. Reengagement behaviour per se could thus best be interpreted as a necessary - but not sufficient - condition of joint commitment. More certain evidence of joint commitment could be gathered by having apes play in parallel with a human experimenter and compare the reengagement behaviours between social no-commitment and joint commitment conditions [26,57]. Nonetheless, caution in interpreting reengagement data should equally apply to human children, who are unable to convincingly express commitment at an early age. In studies involving very young children, conclusions are likewise often based on nonverbal behavior, which must be carefully controlled [e.g., 26,30]. Generally, it may be beneficial to not only assess joint commitment based on reengagement behaviours, as classically done. Rather, a more inclusive and holistic comparative analysis is needed, which examines the way in which participants naturally communicate in spontaneous interactions between peers [14,15,18]. Looking at the whole process of joint action coordination, i.e., how apes and humans get into and out of interactions [20,21], or how they repair communicative breakdowns [58], could be particularly fruitful to deliver more representative interaction data.

Since we tested great apes while engaging with human partners rather than with conspecifics, one could also argue that the apes may not express or develop reengagement behaviour under natural conditions. Our related observational research revealed that apes' reengagement attempts extend to conspecifics in naturally occurring social grooming and play activities, at least in captivity [24,32]. However, beyond anecdotal evidence [p. 123 [59]], it remains unclear whether this ability is specific to captive groups or extends to wild apes – representing an exciting opportunity for future research. To address whether reengagement behaviour is specific to *Pan* or *Homo*, further studies might additionally apply this paradigm to other primate species, or animal taxa more distant from humans. One recent study has already shown reengagement behaviour in dogs [60], pointing to the possibility of convergent evolutionary origins in relation to joint commitment.

Another limitation is that the apes interacted with multiple experimenters with whom they had varying levels of familiarity. Social relationships are important for apes [20], though partner choice in cooperative interactions may differ between species. For instance, bonobos generally appear less selective in their choice of partners in cooperative contexts compared to chimpanzees [see discussions in 60]. While the cited studies examined social bonds between conspecifics, the quality of relationships likely also varies in interactions with human partners [61]. The apes in our study interacted both with human caregivers (i.e., keepers and surrogate mothers) and researchers, with varying degrees of familiarity, likely exhibiting closer attachments with caregivers and weaker ones with researchers. These differences in familiarity may have influenced reengagement rates. However, our findings indicated that familiarity levels with human experimenters did not appear to affect reengagement rates [a similar lack of effect of social bond on reengagement rate was also reported between conspecifics in bonobos and chimpanzees in 32]. Despite this null finding, the role of relationship quality in shaping reengagement behavior still warrants further investigation in future studies, whether in interactions with conspecifics or humans.

Although it is often assumed that bonobos are more socially tolerant and pro-social than chimpanzees [40,41], we did not find differences in any of the assessed behaviours between the two groups. In line with another study that compared reengagement rates in natural joint actions of bonobos and chimpanzees [32], our data revealed similar reengagement and signalling rates between the two groups, suggesting that our bonobo subjects were not necessarily

more motivated to reconstruct previous commitments with others than were chimpanzees. Our data fits with previous studies on joint commitment, which have shown that chimpanzees, as much as bonobos, appear to exhibit behavioural correlates indicative of their potential engagement in joint commitment [34]. The current findings, along with others [20,24,32], point to a continuous evolution of joint commitment, with the early foundation likely having evolved earlier than previously assumed [10], either with (or before) our last common ancestor with *Pan*, or as a convergent adaptation to the demands of joint action coordination. Yet, further controls and data from more groups and wild settings need to be included before firm conclusions can be drawn.

Lastly, in contrast to our prediction, we found no statistical evidence for differences in reengagement behaviours across conditions in either group. One reason for this may be our small sample size; we were only able to observe trends, notably in adult bonobos, who produced slightly more communicative signals and GRBs when experimenters were facing towards them than when they were facing away. Firm conclusions cannot be drawn from these limited analyses and should be expanded in further studies with more comprehensive sample sizes and conditions of differing intentions of the experimenter. For instance, one might add conditions resembling those used in [62], by having experimenters who are willing to reengage (but unable) or are unwilling to reengage (but able).

Conclusions

Our findings showed that chimpanzees and bonobos, even at a young age, have the propensity to reengage a passive partner to a triadic joint game after an interruption. However, infant bonobos communicated less during interruption phases compared to adult bonobos, yielding weaker evidence of reengagement in younger subjects. Reengagement attempts of young bonobos also mainly contained tactile gestures or signals unrelated to the game, while reengagement attempts in adult bonobos (and chimpanzees) often comprised GRBs, indicating a potentially more sophisticated understanding of the joint game in adults. Future studies should attempt to further pinpoint fine-grained differences in behavioural manifestations of joint commitments in humans and other primates via a bottom-up-approach, investigating all kinds of behaviours and signals, as well as bodily movements not necessarily classified as signals [63]. Although reengagement represents one behavioural correlate of joint commitment, we advocate future studies to study additional components, such as signal exchanges to coordinate entries and exit of joint activities [14,15].

Supporting information

S1 Text. Additional attempted yet failed conditions.
(DOCX)

S1 Table. Information about study subjects.
(DOCX)

S2 Table. Ethogram of signals (i.e., gestures, vocalizations, facial expressions) and GRBs.
(DOCX)

S3 Table. Count of signals and game-related behaviours (GRB) used to reengage the partner across groups .
(DOCX)

S1 Data. Datasets supporting the analyses of this paper.
(PDF)

Acknowledgments

We thank La Vallée des Singes and its former director Emmanuel Le Grelle as well as Le Conservatoire pour la Conservation des Primates and ABC Congo, particularly Fanny Minesi, Raphaël Belais and Claudine André, for allowing access to the study sites and subjects. We are particularly thankful for the support of the animal keepers at La Vallée des Singes and at Lola Ya Bonobo who acted as experimenters and helped us implement the trials, and to Héritier Izansone, for his invaluable assistance throughout the study.

Author contributions

Conceptualization: Raphaela Heesen, Adrian Bangerter, Klaus Zuberbühler, Federico Rossano, Emilie Genty.

Data curation: Raphaela Heesen, Emilie Genty.

Formal analysis: Raphaela Heesen, Katia Iglesias.

Funding acquisition: Adrian Bangerter, Klaus Zuberbühler.

Investigation: Raphaela Heesen, Emilie Genty.

Methodology: Raphaela Heesen, Federico Rossano, Jean-Pascal Guéry, Emilie Genty.

Project administration: Emilie Genty.

Resources: Jean-Pascal Guéry.

Supervision: Adrian Bangerter, Klaus Zuberbühler, Emilie Genty.

Validation: Katia Iglesias, Emilie Genty.

Visualization: Raphaela Heesen.

Writing – original draft: Raphaela Heesen.

Writing – review & editing: Raphaela Heesen, Adrian Bangerter, Klaus Zuberbühler, Katia Iglesias, Federico Rossano, Emilie Genty.

References

1. Boesch C. Joint cooperative hunting among wild chimpanzees: Taking natural observations seriously. Behav Brain Sci. 2005;28(5):692–3. https://doi.org/10.1017/s0140525x05230121
2. Holekamp KE, Sakai ST, Lundrigan BL. The Spotted Hyena (Crocuta crocuta) as a Model System for Study of the Evolution of Intelligence. Journal of Mammalogy. 2007;88(3):545–54. https://doi.org/10.1644/06-mamm-s-361r1.1
3. Pitman RL, Durban JW. Cooperative hunting behavior, prey selectivity and prey handling by pack ice killer whales (Orcinus orca), type B, in Antarctic Peninsula waters. Marine Mammal Science. 2011;28(1):16–36. https://doi.org/10.1111/j.1748-7692.2010.00453.x
4. Vail AL, Manica A, Bshary R. Referential gestures in fish collaborative hunting. Nat Commun. 2013;4:1765. https://doi.org/10.1038/ncomms2781 PMID: 23612306
5. Fonio E, Heyman Y, Boczkowski L, Gelblum A, Kosowski A, Korman A, et al. A locally-blazed ant trail achieves efficient collective navigation despite limited information. Elife. 2016;5:e20185. https://doi.org/10.7554/eLife.20185 PMID: 27815944
6. McCreery HF, Dix ZA, Breed MD, Nagpal R. Collective strategy for obstacle navigation during cooperative transport by ants. J Exp Biol. 2016;219(Pt 21):3366–75. https://doi.org/10.1242/jeb.143818 PMID: 27807216
7. Clutton-Brock T. Cooperation between non-kin in animal societies. Nature. 2009;462(7269):51–7. https://doi.org/10.1038/nature08366 PMID: 19890322
8. Call J. Contrasting the social cognition of humans and nonhuman apes: the shared intentionality hypothesis. Top Cogn Sci. 2009;1(2):368–79. https://doi.org/10.1111/j.1756-8765.2009.01025.x PMID: 25164939

9. Tomasello M, Carpenter M. Shared intentionality. Dev Sci. 2007;10(1):121–5. https://doi.org/10.1111/j.1467-7687.2007.00573.x PMID: 17181709

10. Tomasello M. A natural history of human morality. Cambridge, Massachusetts: Harvard University Press; 2016.

11. Clark HH. Social actions, social commitments. In: Enfield NJ, Levinson SC, editors. Roots of human sociality: Culture, cognition and interaction. Oxford, England: Berg; 2006. pp. 126–150.

12. Gilbert M. Joint commitment. In: Jankovic M, Ludwig K, editors. The Routledge handbook of collective intentionality. New York: Routledge; 2017. pp. 130–139.

13. Tomasello M, Moll H. The gap is social: human shared intentionality and culture. In: Kappeler PM, Silk JB, editors. Mind the gap Tracing the origins of human universals. Berlin: Springer; 2010. pp. 331–349.

14. Bangerter A, Genty E, Heesen R, Rossano F, Zuberbühler K. Every product needs a process: unpacking joint commitment as a process across species. Philos Trans R Soc Lond B Biol Sci. 2022;377(1859):20210095. https://doi.org/10.1098/rstb.2021.0095 PMID: 35876205

15. Heesen R, Genty E, Rossano F, Zuberbühler K, Bangerter A. Social play as joint action: A framework to study the evolution of shared intentionality as an interactional achievement. Learn Behav. 2017;45(4):390–405. https://doi.org/10.3758/s13420-017-0287-9 PMID: 28779386

16. Leavens DA, Bard KA, Hopkins WD. The mismeasure of ape social cognition. Anim Cogn. 2019;22(4):487–504. https://doi.org/10.1007/s10071-017-1119-1 PMID: 28779278

17. Clark HH. Using language. Cambridge: Cambridge University Press; 1996.

18. Genty E, Heesen R, Guéry J-P, Rossano F, Zuberbühler K, Bangerter A. How apes get into and out of joint actions: precursors of shared. Interact Stud. 2020;21(3):353–86. https://doi.org/10.1075/is.18048.gen

19. Gilbert M. Obligation and Joint Commitment. Utilitas. 1999;11(2):143–63. https://doi.org/10.1017/s0953820800002399

20. Heesen R, Bangerter A, Zuberbühler K, Iglesias K, Neumann C, Pajot A, et al. Assessing joint commitment as a process in great apes. iScience. 2021;24(8):102872. https://doi.org/10.1016/j.isci.2021.102872 PMID: 34471860

21. Rossano F, Terwilliger J, Bangerter A, Genty E, Heesen R, Zuberbühler K. How 2- and 4-year-old children coordinate social interactions with peers. Philos Trans R Soc Lond B Biol Sci. 2022;377(1859):20210100. https://doi.org/10.1098/rstb.2021.0100 PMID: 35876200

22. Chevalley E, Bangerter A. Suspending and Reinstating Joint Activities With Dialogue. Discourse Processes. 2010;47(4):263–91. https://doi.org/10.1080/01638530902959935

23. Gräfenhain M, Carpenter M, Tomasello M. Three-year-olds' understanding of the consequences of joint commitments. PLoS One. 2013;8(9):e73039. https://doi.org/10.1371/journal.pone.0073039 PMID: 24023805

24. Heesen R, Bangerter A, Zuberbühler K, Rossano F, Iglesias K, Guéry J-P, et al. Bonobos engage in joint commitment. Sci Adv. 2020;6(51):eabd1306. https://doi.org/10.1126/sciadv.abd1306 PMID: 33355132

25. Gilbert M. Walking Together: A paradigmatic social phenomenon. Midwest Studies in Philosophy. 1990;15:1–14. https://doi.org/10.1111/j.1475-4975.1990.tb00202.x

26. Gräfenhain M, Behne T, Carpenter M, Tomasello M. Young children's understanding of joint commitments. Dev Psychol. 2009;45(5):1430–43. https://doi.org/10.1037/a0016122 PMID: 19702403

27. Kachel U, Svetlova M, Tomasello M. Three-Year-Olds' Reactions to a Partner's Failure to Perform Her Role in a Joint Commitment. Child Dev. 2018;89(5):1691–703. https://doi.org/10.1111/cdev.12816 PMID: 28508396

28. Kachel U, Svetlova M, Tomasello M. Three- and 5-year-old children's understanding of how to dissolve a joint commitment. J Exp Child Psychol. 2019;18434–47. https://doi.org/10.1016/j.jecp.2019.03.008 PMID: 30999155

29. Kachel U, Tomasello M. 3- and 5-year-old children's adherence to explicit and implicit joint commitments. Dev Psychol. 2019;55(1):80–8. https://doi.org/10.1037/dev0000632 PMID: 30359057

30. Warneken F, Chen F, Tomasello M. Cooperative activities in young children and chimpanzees. Child Dev. 2006;77(3):640–63. https://doi.org/10.1111/j.1467-8624.2006.00895.x PMID: 16686793

31. Tomasello M. Becoming human: A theory of ontogeny. Cambridge, Massachusetts: Harvard University Press; 2019.

32. Heesen R, Zuberbühler K, Bangerter A, Iglesias K, Rossano F, Pajot A, et al. Evidence of joint commitment in great apes' natural joint actions. R Soc Open Sci. 2021;8(12):211121. https://doi.org/10.1098/rsos.211121 PMID: 34909217
33. Hirata S, Fuwa K. Chimpanzees (*Pan troglodytes*) learn to act with other individuals in a cooperative task. Primates. 2007;48(1):13–21. https://doi.org/10.1007/s10329-006-0022-1 PMID: 17103081
34. MacLean E, Hare B. Spontaneous triadic engagement in bonobos (*Pan paniscus*) and chimpanzees (*Pan troglodytes*). J Comp Psychol. 2013;127(3):245–55. https://doi.org/10.1037/a0030935 PMID: 23339560
35. Martin CF, Biro D, Matsuzawa T. Chimpanzees spontaneously take turns in a shared serial ordering task. Sci Rep. 2017;7(1):14307. https://doi.org/10.1038/s41598-017-14393-x PMID: 29093539
36. Pika S, Zuberbühler K. Social games between bonobos and humans: evidence for shared intentionality?. Am J Primatol. 2008;70(3):207–10. https://doi.org/10.1002/ajp.20469 PMID: 17894382
37. Voinov PV, Call J, Knoblich G, Oshkina M, Allritz M. Chimpanzee Coordination and Potential Communication in a Two-touchscreen Turn-taking Game. Sci Rep. 2020;10(1):3400. https://doi.org/10.1038/s41598-020-60307-9 PMID: 32098996
38. Gruber T. Wild-Born Orangutans (Pongo abelii) Engage in Triadic Interactions During Play. Int J Primatol. 2013;35(2):411–24. https://doi.org/10.1007/s10764-013-9745-1
39. Gruber T, Clay Z. A Comparison Between Bonobos and Chimpanzees: A Review and Update. Evol Anthropol. 2016;25(5):239–52. https://doi.org/10.1002/evan.21501 PMID: 27753219
40. Hare B, Melis AP, Woods V, Hastings S, Wrangham R. Tolerance allows bonobos to outperform chimpanzees on a cooperative task. Curr Biol. 2007;17(7):619–23. https://doi.org/10.1016/j.cub.2007.02.040 PMID: 17346970
41. Tan J, Ariely D, Hare B. Bonobos respond prosocially toward members of other groups. Sci Rep. 2017;7(1):14733. https://doi.org/10.1038/s41598-017-15320-w PMID: 29116154
42. Jaeggi AV, Stevens JMG, Van Schaik CP. Tolerant food sharing and reciprocity is precluded by despotism among bonobos but not chimpanzees. Am J Phys Anthropol. 2010;143(1):41–51. https://doi.org/10.1002/ajpa.21288 PMID: 20310060
43. Iki S, Hasegawa T. Face-to-face configuration in Japanese macaques functions as a platform to establish mutual engagement in social play. Anim Cogn. 2021;24(6):1179–89. https://doi.org/10.1007/s10071-021-01508-1
44. Siposova B, Tomasello M, Carpenter M. Communicative eye contact signals a commitment to cooperate for young children. Cognition. 2018;179:192–201. https://doi.org/10.1016/j.cognition.2018.06.010 PMID: 29960903
45. Wittenburg P, Brugman H, Russel A, Klassmann A, Sloetjes H. ELAN: a professional framework for multimodality research. Proceedings of the 5th International Conference on Language Resources and Evaluation (LREC 2006). 2006;1556–9.
46. Byrne RW, Cartmill E, Genty E, Graham KE, Hobaiter C, Tanner J. Great ape gestures: intentional communication with a rich set of innate signals. Anim Cogn. 2017;20(4):755–69. https://doi.org/10.1007/s10071-017-1096-4 PMID: 28502063
47. Landis JR, Koch GG. The Measurement of Observer Agreement for Categorical Data. Biometrics. 1977;33(1):159. https://doi.org/10.2307/2529310
48. Field A, Miles J, Field Z. Discovering statistics using R. London: Sage; 2012.
49. Leavens DA, Hopkins WD. Intentional communication by chimpanzees: a cross-sectional study of the use of referential gestures. Dev Psychol. 1998;34(5):813–22. https://doi.org/10.1037//0012-1649.34.5.813 PMID: 9779730
50. Furuichi T, Idani G, Ihobe H, Kuroda S, Kitamura K, Mori A, et al. Population dynamics of wild bonobos Pan paniscus at Wamba. International Journal of Primatology. 1998;19(6):1029–43. https://doi.org/10.1023/a:1020326304074
51. Fröhlich M. Taking turns across channels: Conversation-analytic tools in animal communication. Neurosci Biobehav Rev. 2017;80:201–9. https://doi.org/10.1016/j.neubiorev.2017.05.005 PMID: 28501552
52. Fröhlich M, Wittig RM, Pika S. The ontogeny of intentional communication in chimpanzees in the wild. Dev Sci. 2019;22(1):e12716. https://doi.org/10.1111/desc.12716 PMID: 30156360
53. Eckerman CO, Peterman K. Peers and infant social/communicative development. In: Bremner G, Fogel A, editors. Blackwell handbook of infant development. Oxford, UK: Blackwell Publishing Ltd; 2001. pp. 326–350.
54. Hamann K, Warneken F, Tomasello M. Children's developing commitments to joint goals. Child Dev. 2012;83(1):137–45. https://doi.org/10.1111/j.1467-8624.2011.01695.x PMID: 22172281

55. Bard KA, Bakeman R, Boysen ST, Leavens DA. Emotional engagements predict and enhance social cognition in young chimpanzees. Dev Sci. 2014;17(5):682–96. https://doi.org/10.1111/desc.12145 PMID: 24410843

56. Bard KA, Leavens DA. The Importance of Development for Comparative Primatology. Annu Rev Anthropol. 2014;43(1):183–200. https://doi.org/10.1146/annurev-anthro-102313-030223

57. Tomasello M. The coordination of attention and action in great apes and humans. Philos Trans R Soc Lond B Biol Sci. 2022;377(1859):20210093. https://doi.org/10.1098/rstb.2021.0093 PMID: 35876209

58. Heesen R, Fröhlich M, Sievers C, Woensdregt M, Dingemanse M. Coordinating social action: a primer for the cross-species investigation of communicative repair. Philos Trans R Soc Lond B Biol Sci. 2022;377(1859):20210110. https://doi.org/10.1098/rstb.2021.0110 PMID: 35876201

59. Plooij F. Some basic traits of language in wild chimpanzees? In: Lock A, editor. Gesture and Symbol: The Emergence of Language. London, UK: Academic Press; 1978. pp. 111–131.

60. Horschler DJ, Bray EE, Gnanadesikan GE, Byrne M, Levy KM, Kennedy BS, et al. Dogs re-engage human partners when joint social play is interrupted: a behavioural signature of shared intentionality?. Animal Behaviour. 2022;183159–68. https://doi.org/10.1016/j.anbehav.2021.11.007

61. Genty E, Neumann C, Zuberbühler K. Bonobos modify communication signals according to recipient familiarity. Sci Rep. 2015;5:16442. https://doi.org/10.1038/srep16442 PMID: 26552655

62. Call J, Hare B, Carpenter M, Tomasello M. "Unwilling" versus "unable": chimpanzees' understanding of human intentional action. Dev Sci. 2004;7(4):488–98. https://doi.org/10.1111/j.1467-7687.2004.00368.x PMID: 15484596

63. van Boekholt B, Wilkinson R, Pika S. Bodies at play: the role of intercorporeality and bodily affordances in coordinating social play in chimpanzees in the wild. Front Psychol. 2024;14:1206497. https://doi.org/10.3389/fpsyg.2023.1206497 PMID: 38292528
